# Memory Monitoring Recognition Test (MMRT), a new measurement of stimular source monitoring: Software and comprehension

**DOI:** 10.1371/journal.pone.0321991

**Published:** 2025-04-28

**Authors:** Pedro C. Martínez-Suárez, José Alejandro Valdevila Figueira, Joselyn M. Luna-Cambi, Carlos E. Guerrero-Granda, Rocío Valdevila Santiesteban

**Affiliations:** 1 Catholic University of Cuenca, Cuenca-Ecuador; 2 Psychology and Psychiatry Research Group (GIPSI), Ecuador; 3 Institute of Neurosciences of Guayaquil, Guayaquil-Ecuador; 4 Ecotec University, Guayaquil-Ecuador; Institute of Theoretical and Applied Informatics Polish Academy of Sciences: Instytut Informatyki Teoretycznej i Stosowanej Polskiej Akademii Nauk, UKRAINE

## Abstract

**Background:**

Reality monitoring allows the evaluation and monitoring of reality through the assignment of information to internal or external sources, which is crucial to differentiate real events from imaginary ones. In schizophrenics, monitoring seems to be related to an error in the allocation processes, giving rise to false perceptions such as visual hallucinations, which are associated with a poor prognosis. This error can appear almost imperceptibly at an early age in life, making carrying out predictive or evaluation tests with paper and pencil unattractive. The computerization of technical resources that allow the monitoring of reality offers a new tool to evaluate the attribution process, in an effective and agile way and with easy understanding of cognitive deficits in a friendly environment.

**Objective:**

Computerize the Memory Monitoring and Recognition Test (MMRT) evaluate reality monitoring through verbal memory tasks, improving its implementation, optimizing interaction with the user and perfecting the recording of memory errors that could indicate psychotic symptoms.

**Method:**

The MMRT was developed using Python and Kivy, facilitating the creation of cross-platform user interfaces. The test is structured in stages, allows voice accessibility for people with visual disabilities and provides comprehensive user management. The test data is stored in the cloud using MongoDB as the database system. Additionally, the software incorporates speech recognition using the gTTS library and generates a performance report in PDF format, documenting external, internal and global attribution errors.

**Result:**

The computerized version of the MMRT allowed the detection of specific errors in memory monitoring, as well as the performance of repeated measurements to evaluate long-term memory and working memory.

**Conclusion:**

Preliminary applications suggest its usefulness in identifying early cognitive markers of schizophrenia, facilitating the measurement of reality monitoring through attribution errors. Developed with open-source technology and an interface adaptable to various platforms, the MMRT represents an accessible and efficient tool for psychological evaluation, with innovative potential in the study of reality monitoring.

## Introduction

Schizophrenia is a serious and disabling psychiatric disorder [1,2], which affects all social classes and ethnic groups globally [3]. Its evolutionary course is progressive and deteriorating, depending on the age of onset and the clinical form of presentation. It occurs with a higher incidence in early adulthood, which contributes to the appearance of severe cognitive deficits in the medium and long term [4]. Schizophrenia evolves through outbreaks and relapses; in addition, there are cognitive biases that may be involved in the onset, maintenance and relapses [5].

Between 70–75% of patients with schizophrenia show lower performance than the general population in cognitive tasks [6,7], with memory being the most affected process. Furthermore, there is a high correlation between the nature of cognitive impairment in reality processing and its relationship with the positive symptoms of schizophrenia [8,9].

Memory disturbances are often due to errors in identifying the origin of mental experiences, such as failures in reality monitoring and incorrect source assignments [5]. This manifests itself as a confusion between what the subject imagined and what really happened. Patients mix what they observe directly with what is suggested to them, exchanging the actions they perceived with what they heard or already knew, without being able to distinguish between what is fictitious and what is real, which is understood as an error in identifying the source. These errors occur because the information retrieved is often partial or ambiguous, and the processes responsible for assigning this information to its original sources have failed [10].

The assignment failure occurs due to a fragmentation of short-term memory, which makes it difficult to retain, manipulate and integrate sensory information and form a coherent representation of reality, according to Johnson and Raye [8,9], explained through different theoretical models [11,12,13].

Information processing tests are used as markers of vulnerability to cognitive errors in order to identify psychotic symptoms in subjects with difficulties in performing source monitoring and information attribution tests [14,15,16]. The exploration of cognitive tasks to measure reality monitoring has been carried out mostly with tests in pencil and paper format [17,18], in english [19] and without considering the special requirements of some patients [20].

The tests used, during reality monitoring, explore conscious retrospective judgments about the source of information and self-agency judgments of each subject [21] that have been explored with current technological resources to demonstrate the existence of an externalization bias or erroneous attribution about the source of current memories and thoughts [22] through the temporal evaluation of reality monitoring and impaired self-referential memory, such as the use of Functional Magnetic Resonance Imaging (fMRI) [23].

The MMRT is a verbal cognitive task that involves the structures involved in monitoring reality through verbal feedback, designed by Martínez, Lemos and Paino (1997) [24], complemented with the Ball Control Test (BCT) developed by Martínez (2021) [25], which has a visual and motor component that turns the evaluation into a friendlier process. The possibility of computerizing the MMRT offers an easy-to-apply diagnostic screening tool that, combined with fMRI studies, would allow greater specificity and sensitivity in the identification of the activity of the prefrontal cortex (PFC) in the expression of schizophrenia, offering robustness to the diagnosis of the organic damage that accompanies this pathology and allowing the development of more precise therapeutic strategies.

## Method

Prior to the proposal of the digitalized MMRT model proposed by our research, a paper and pencil study was carried out with a limited sample of clinical cases. The study was divided into two stages, the clinical evaluation in a paper-and-pencil model and the development of a digitized model of the MMRT tool.

The MMRT software was developed to obtain a measurement of reality monitoring, based on a cognitive test that uses a verbal memory component to identify internal and external attribution errors, which are related to psychotic symptomatology for the early identification of schizophrenia.

## First stage

### Participants

Forty patients of the 480 seen in the initial or subsequent consultation between 1994 and 1995 at a health center in Spain were randomly included. The 40 patients had a psychiatric diagnosis according to the current classifier (ICD-10) and were grouped into 4 classes: psychotics with auditory hallucinations, psychotics without auditory hallucinations, affective disorders and neurotic disorders. All classes had 10 participating cases per capita. All were evaluated with MMRT and BCT [25]. Subjects aged between 18 and 22 were included (27 were women). Participants with difficulty understanding the test were excluded. Most patients were diagnosed with schizophrenia (n = 7.18%). They were explained what the test consisted of and the objectives of the study to obtain informed consent, as well as the possibility of withdrawing at any time without consequences.

## Instruments

In this study, we employed two main instruments to assess Memory Monitoring Recognition: The MMRT and the paper-and-pencil BCT. The MMRT is a recognition monitoring memory task. This test is particularly valuable for detecting recall of the stimulus source as it assesses source monitoring memory. For this study, we used a total score calculated from all subscales to capture an overall measure of cognitive performance. The BCT (Balls Control Test) is a tool named for its content (in its standard presentation spheres that move on the screen and of which the subject only controls one of them using a keyboard or control).

In addition, the Medical History provided a detailed clinical record of each participant, including sociodemographic data. Problems in recognition memory monitoring were diagnosed when a participant referred to voices or stimulus sources that were not under his or her conscious control [26].

Participants underwent a psychiatric evaluation using the abbreviated 18-item Brief Psychiatric Rating Scale (BPRS) [27], MMRT [28], 10 cards from the Luria-Nebraska Battery (M test) listed on the recording sheet [29], as well as the 10 words written on cards for the third subtask.

## Second stage

### MMRT digital version

The MMRT is software developed in Python. For the creation of the MMRT, the Python framework called Kivy was used, designed for the development of cross-platform user interfaces (GUI), which offers a friendly interface that can be installed on any modern laptop, facilitating the application of the verbal cognitive task focused on memory, for the measurement of reality monitoring.

**Fig 1:**
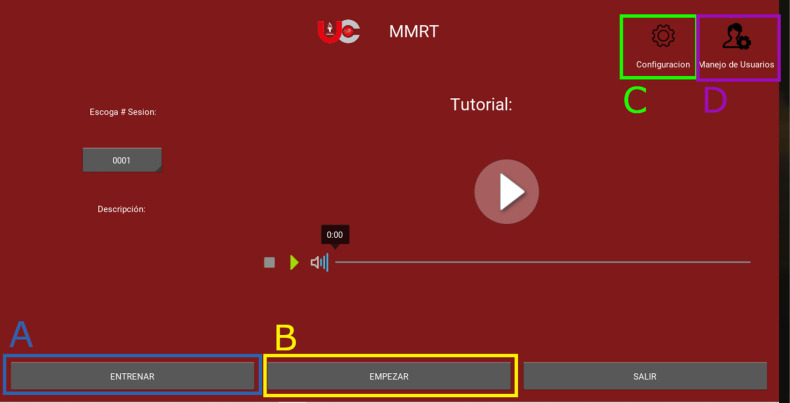
Memory monitoring recognition test main screen.

**Fig 2:**
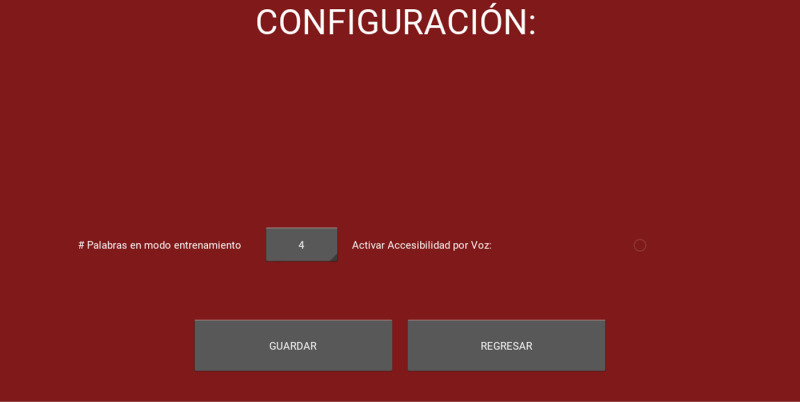
Memory monitoring recognition test general settings.

**Fig 3:**
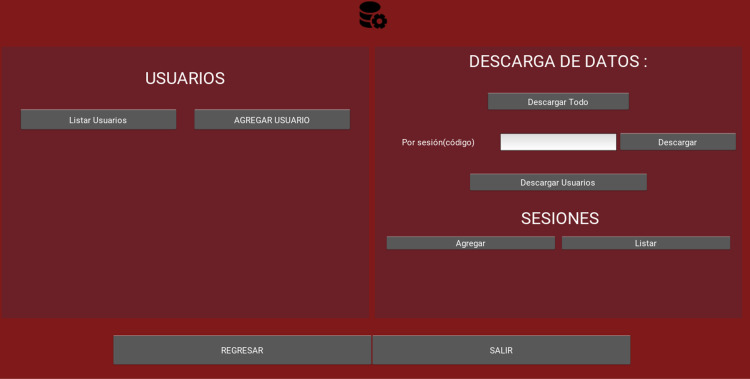
Memory monitoring recognition test admin settings.

**The MMRT main screen is divided into four components (** **Fig 1** **):**

(A) **Training section:** In this window a simulation of the main test is carried out, in which words other than those used in the main evaluation are presented to avoid contamination of the results. The data generated in this section is not stored, as its purpose is to familiarize the user with the test and its format.
(B) **Main test section** In this window the test is carried out (Fig 3). As a first step, the previously registered user must select their profile. The search is performed using a list of users by first or last name. At the end of the test, the data obtained is saved in the cloud and a PDF report is generated with the user’s data and the corresponding metrics.
(C) **Configuration section:** This section contains the general test settings (Fig 2).

**Number of Training Words** Allows you to select the number of words to display in the training section.

**Enable voice accessibility** allows you to activate voice assistance so that the test can be administered to subjects with visual disabilities.

**Fig 4:**
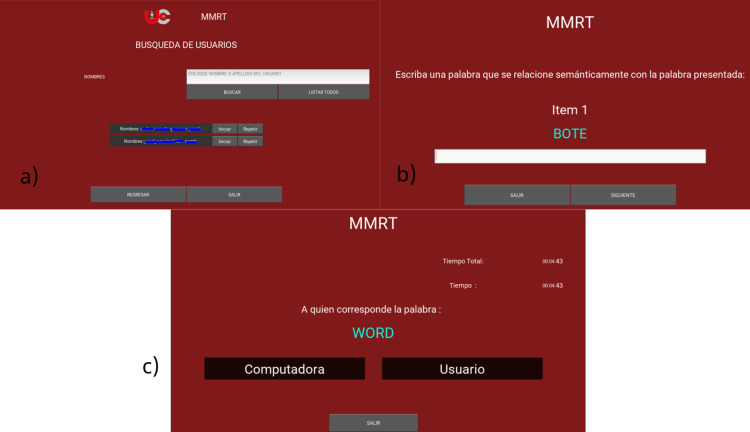
Memory monitoring recognition test main test screen.

Additionally, this same section allows you to add, edit or delete the profiles of the users who take the test (Fig 4). This window contains options to download a CSV file with a summary of all tests performed. It also includes the option to add a brief description of the populations to which the test can be applied.

(D) **Database administration section.**

Resources such as Kivy, pandas, gTTS (Google Text-to-Speech), pdfkit and jinja2 were used to develop the software. Additionally, the software backs up all test results to the cloud using MongoDB, a non-relational database.

## Home and voice accessibility

To start a test, the user must select a profile or create one in the Administration tab. Subsequently, within the administration window, the user profile must be created. Once these requirements are met, the test can begin.

Voice accessibility is disabled by default. If necessary, it can be activated in the settings window. On the screen, the profile of the user who will perform the test is searched and selected. A consent document is then presented, which the patient must read and accept before proceeding with the evaluation.

## First stage procedure

For the clinical evaluation, data were collected from the clinical records of initial care and/or follow-up of subjects previously diagnosed as psychotic with auditory hallucinations, psychotic without auditory hallucinations, affective disorders and neurotic disorders, stored in the Department of Archives and Statistics of the Health Service of the Principality of Asturias (SESPA). An Excel database was created to facilitate analysis and improve understanding. The search focused on identifying the pathologies mentioned during the preparation of the clinical history.

## Second stage procedure

Once the test has started, words are shown to the user, who must write a word related to the one presented or mention it out loud, if voice accessibility is enabled. When all the answers from the first phase have been saved, they are mixed with the questions and a new list of questions is generated. In this second phase, the user is asked to select which one they believe has been sent by: (A) the computer or, (B) the user. These are the only accepted answers. During this process, the reaction time until the user selects an option is measured. At the end of all the questions in the second phase, a PDF report is generated with the answers, reaction times and the total errors made during the test.

## Long-term memory assessment

To assess long-term memorization, the software includes the option to repeat the test days later. This option is activated when the user completes the test for the first time. Then, when searching for a user who has previously taken the test, the “Repeat” button is enabled. Once this option is selected, the second phase of the test is executed directly, using the answers from the previous attempt.

### Main test flowchart.

**Fig 5:**
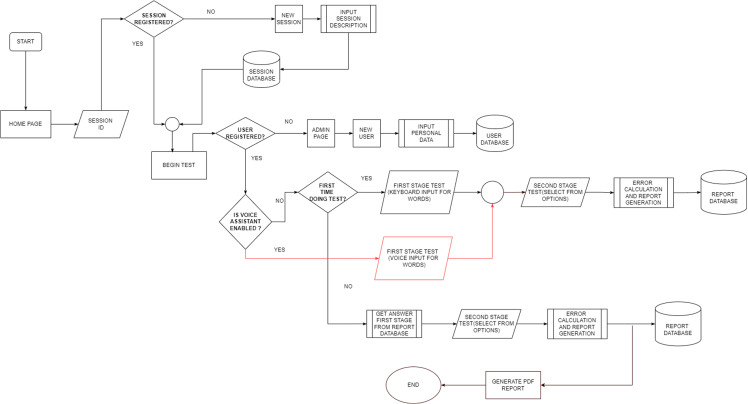
Memory monitoring recognition test workflow.

Fig 5 shows the implemented speech recognition procedure. The gTTS library was carefully selected for use in the programming process as it offers the highest reliability in word detection and is free to use.

When the voice accessibility option is activated, each of the test words is played over the speakers, and a sound indicates that the user’s response is expected. Once the response is obtained, it is converted to text and stored. In the second phase of the test, all the words in the questions and answers are mixed, generating a random sample for each user. Similar to the first phase, each word will be played over the speakers, allowing the user to select between two options: “Computer”, if they believe the word was initially mentioned by the system, or “User”, if they believe the word was initially mentioned by themselves. These are the only accepted answers.

## Labeling process

The labeling process classifies attribution errors into three main categories. 1) External attribution errors that occur when a word written or mentioned by the computer is wrongly attributed by the user as their own, 2) internal attribution errors that occur when a word written or mentioned by the user is incorrectly attributed to the computer, and 3) global errors that encompass both types of attribution errors.

**Fig 6:**
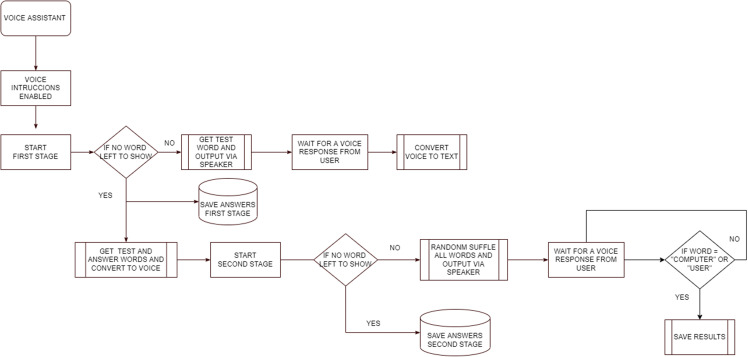
Voice recognition workflow.

For the operation of voice recognition (Fig 6) and the backup of the data obtained in the cloud, an Internet connection is required. This functionality allows multiple instances of the software to run simultaneously, optimizing the application of the test to multiple users at the same time. Being developed in Python, the software is cross-platform. Additionally, for ease of use, an installer for Microsoft Windows was created using the Inno Setup tool.

## Analysis

In the statistical analysis, descriptive tests were used with the objective of characterizing the sociodemographic variables of the sample. Frequencies and percentages were calculated to describe the distribution of the categories, as well as measures of central tendency and dispersion, including the mean, median, standard deviation, minimum and maximum values.

To evaluate the distribution of the data, normality tests were performed using the Shapiro-Wilk test. Likewise, the skewness and kurtosis coefficients were calculated in order to determine if the data fit a normal distribution.

In the inferential analysis, different statistical tests were applied depending on the nature of the variables and the distribution of the data. For the comparison of means between groups defined by sex and age group, analysis of variance (ANOVA) was used, considering their respective significance values (p-value). However, due to the size of the sample and the possible lack of normality in some variables, non-parametric tests were used to guarantee the validity of the results. In this sense, the Wilcoxon test was used to compare medians between two groups, while the Kruskal-Wallis test was applied to compare more than two groups. These tests allowed us to analyze differences between groups without assuming a normal distribution of the data.

Finally, the analysis was complemented with the interpretation of the statistics obtained, evaluating the significance of the results and their implication in the understanding of the variables studied. Furthermore, for a better description of the dispersion of the data in non-parametric variables, interquartile ranges (Q125% - Q375%) were calculated, providing additional information on the variability of the data and the presence of outliers. All statistical analyzes in this study were performed with the SPSS statistical package (v.5.01 for Windows) [30].

## Results

### Sample characteristics

The sample consisted of 40 participants, of which the majority were women (68%, n = 27), while men represented 32% (n = 13). The age group 20–22 years predominated (60%, n = 24) followed by the group 18–19 (40%, n = 16). 42% (n = 17) of the participants had a primary education level, 40% (n = 16) completed high school, 7.5% (n = 3) had university studies, and 10% (n = 4) did not have formal education. 48% (n = 19) of participants were married, 32% (n = 13) were single, 15% (n = 6) were separated or divorced, and 5% (n = 2) were widowed. The majority of participants (90%, n = 36) were inactive at work. Regarding the clinical condition of the participants, an equitable distribution was evident between the four established groups (10 participants) with 25% (n = 10) for each: 1. psychosis and hallucinations, 2. psychosis without hallucinations, 3. affective disorders and 4. neurotic disorders. The most common diagnosis was schizophrenia (18%, n = 7), followed by recurrent depressive disorder (12%, n = 5) and delusional disorder (12%, n = 5). Other diagnoses noted included dysthymia (10%, n = 4), hallucinatory disorders (10%, n = 4), somatoform disorder (7.5%, n = 3), and schizoaffective disorder (7.5%, n = 3). The least frequent diagnoses were bipolar disorder, paraphrenia, adjustment disorder and dissociative disorder with one case per capita and anxiety disorder with 2 cases (5%) (Table 1).

**Table 1:** Sociodemographic variables, conditions and diagnosis.

Characteristics	N = 40
**Sex**	
Woman	27 (68%)
Man	13 (32%)
**Age group**	
18-19	16 (40%)
20-22	24 (60%)
**Educational level**	
Bachelor	16 (40%)
Primary	17 (42%)
Without studies	4 (10%)
University	3 (7.5%)
**Marital status**	
Married	19 (48%)
Separated or divorced	6 (15%)
Single	13 (32%)
Widower	2 (5.0%)
**Employment status**	
Asset	4 (10%)
Idle	36 (90%)
**Condition**	
Group 1 (psychotics with hallucinations)	10 (25%)
Group 2 (psychotics without hallucinations)	10 (22.5%)
Group 3 (affective disorders)	10 (25%)
Group 4 (neurotic disorders)	10 (25%)
**Composition by diagnosis**	
Hallucinatory disorder	4 (10%)
Bipolar disorder	1 (2.5%)
Recurrent depression	5 (12%)
Dysthymia	4 (10%)
Schizoaffective disorder	3 (7.5%)
Schizophrenia	7 (18%)
Paraphrenia	1 (2.5%)
Rent	3 (7.5%)
Somatoform disorder	3 (7.5%)
Delusional disorder	5 (12%)
Adaptive disorder	1 (2.5%)
Anxiety disorder	2 (5.0%)
Dissociative disorder	1 (2.5%)
^*1*^ *n (%)*	

The results show that the total score of GE (General Errors) obtained a mean of 11.00 and a median of the same value (SD = 0.7161), which indicates a low variability in the responses. The minimum and maximum values range between 10.00 and 12.00, and the skewness is 0.0000, suggesting a symmetric distribution. The negative kurtosis (-1.31) indicated a flatter than normal distribution. The Shapiro-Wilk test yields a value of W = 0.889 and p <.001, indicating a significant deviation from normality.

The total IAE score (Internal Attribution Errors) had a mean of 5.29 and a median of 5.30 (SD = 0.2495). Its minimum and maximum values were 5.00-5.60, indicating low dispersion. The skewness was 0.0480, close to symmetry, but the kurtosis of -1.56 suggests a flatter distribution. The normality test (W = 0.792, p <.001) confirmed that the distribution is not normal.

For the total EAE (External Attribution Errors) score, the mean and median are 3.60 (SD = 0.4529). Extreme values range between 3.00 and 4.20. The distribution was approximately symmetric (skewness ≈ 0), but with a negative kurtosis of -1.38. The Shapiro-Wilk test (W = 0.857, p <.001) also indicates a significant deviation from normality.

Regarding the specific tasks, it is observed that GE in Task 1 obtained a mean and median of 4.10 (SD = 0.1013), indicating little dispersion. The skewness was practically zero, but the kurtosis of -2.11 indicated an even flatter distribution. The normality test showed W = 0.637 and p <.001, which confirms a non-normal distribution.

For the IAEs and EAEs in Task 1, the results showed similar trends, with flattened distributions and significant normality tests. The IAE in Task 1 had a mean of 2.05 and a curtosis of -2.11, while the EAE had a mean of 1.10 and a curtosis of -1.56, both with non-normal distributions.

The data from Task 2 show that GE had a mean of 3.45 and a median of the same value (SD = 0.3397). The skewness was practically zero, and the kurtosis of -1.38 indicated a flattened distribution. The Shapiro-Wilk test (W = 0.857, p <.001) showed that the distribution was not normal. The IAEs and EAEs in this task had similar distributions, with negative curtosis and Shapiro-Wilk values indicating deviation from normality.

Finally, in Task 3, GE had a mean of 4.39 and a median of 4.40 (SD = 0.3327). The skewness was minimal (0.0480) and the curtosis of -1.56 suggests that there was a flatter distribution. The IAE and EAE in this task followed the same pattern of flattened distribution and significant normality tests (Table 2).

**Table 2:** Task descriptions.

							Asymmetry		Curtosis		Shapiro-Wilk		Percentiles		
	**N**	**Mean**	**Median**	**SD**	**Mín.**	**Máx**	**Asymmetry**	**EE**	**Curtosis**	**EE**	**W**	**p**	**25th**	**50th**	**75th**
GE Total	40	11.00	11.00	0.7161	10.00	12.00	0.0000	0.374	-1.31	0.733	0.889	< .001	10.50	11.00	11.50
IAE Total	40	5.29	5.30	0.2495	5.00	5.60	0.0480	0.374	-1.56	0.733	0.792	< .001	5.00	5.30	5.60
EAE Total	40	3.60	3.60	0.4529	3.00	4.20	-1.56e−16	0.374	-1.38	0.733	0.857	< .001	3.30	3.60	3.90
GE Task 1	40	4.10	4.10	0.1013	4.00	4.20	1.39e-14	0.374	-2.11	0.733	0.637	< .001	4.00	4.10	4.20
IAE Task 1	40	2.05	2.05	0.0506	2.00	2.10	1.39e-14	0.374	-2.11	0.733	0.637	< .001	2.00	2.05	2.10
EAE Task 1	40	1.10	1.10	0.0832	1.00	1.20	0.0480	0.374	-1.56	0.733	0.792	< .001	1.00	1.10	1.20
GE Task 2	40	3.45	3.45	0.3397	3.00	3.90	-2.02e−15	0.374	-1.38	0.733	0.857	< .001	3.22	3.45	3.68
IAE Task 2	40	1.20	1.20	0.1663	1.00	1.40	0.0480	0.374	-1.56	0.733	0.792	< .001	1.00	1.20	1.40
EAE Task 2	40	1.70	1.70	0.1432	1.50	1.90	4.94e-16	0.374	-1.31	0.733	0.889	< .001	1.60	1.70	1.80
GE Task 3	40	4.39	4.40	0.3327	4.00	4.80	0.0480	0.374	-1.56	0.733	0.792	< .001	4.00	4.40	4.80
IAE Task 3	40	2.30	2.30	0.2265	2.00	2.60	3.13e-15	0.374	-1.38	0.733	0.857	< .001	2.15	2.30	2.45
EAE Task 3	40	2.40	2.40	0.4297	1.80	3.00	1.01e-15	0.374	-1.31	0.733	0.889	< .001	2.10	2.40	2.70

***Note:*** IAE = internal attribution errors, EAE = external attribution errors.

The results obtained from the analysis of the tasks according to sex and age group (supplementary material 1 in S2 File) found that, in terms of sex, there were no significant differences in the scores for most of the tasks, with the exception of task 1 and task 2 of GE and IAE. For task 1, women presented slightly higher scores than men, with a significant difference (F1,38 = 35.29, P < 0.013), as in task 2 for GE scores (F1,38 = 5.59, P = 0.023).

The age groups showed some differences. For example, for the total GE, the 20–22 year old group showed higher scores than the 18–19 year old group (F1,38 = 114.00, P < 0.013). However, for the IAE and EAE tasks, no significant differences were reported between the groups (F1,38 = 0.07, P = 0.793 for IAE and F1,38 = 0.00, P = 1.003 for EAE in the corresponding tasks).

In general terms, the descriptive analysis of the tasks based on the educational level (supplementary material 2 in S2 File) showed some significant differences, especially in the variables IAE Total, EAE Total, EG Task 2, IAE Task 2, GE Task 3, and IAE Task 3. For the variable IAE Total, a significant difference was observed between the educational level groups (F3,36 = 3.19, P = 0.041). Participants with a Baccalaureate had a slightly lower score than those with Primary, No studies, or University. Similarly, for EAE Total, a significant difference was also recorded (F3,36 = 5.65, P < 0.011), where the Primary group showed a lower score compared to the other groups. Regarding the specific tasks, significant differences were found in GE Task 2 and IAE Task 2, with a p less than 0.011 and 0.041, respectively, indicating that the educational level influences the performance of the participants in these tasks. Specifically, the High School and Primary groups tended to obtain higher scores compared to the No Education and University groups.

In GE Task 3 and IAE Task 3, significant differences were also reported (F3,36 = 3.19, P = 0.041 and F3,36 = 5.65, P < 0.011, respectively), suggesting that educational level has a considerable impact on participants’ performance on these specific tasks.

However, for most tasks in EAE, no significant differences were found between the different educational groups (F3,36 = 0.42, P = 0.741). This indicates that, in terms of EAE, educational level does not have a relevant effect on performance in these tasks.

The marital status of the participants indicated significant and non-significant differences according to the variable analyzed (supplementary material 3 in S2 File). Regarding Total GA, no significant differences were found between the different marital status groups (F3,36 = 0.39, P = 0.761), which suggested that marital status did not influence the total number of general errors committed by the participants. However, for IAE Total, a significant difference was observed (F3,36 = 26.79, P < 0.011). Married and Separated or divorced participants had higher scores than Single and Widowed, which indicated that marital status did influence the number of errors attributed to internal causes. Regarding Total EAE, no significant differences were obtained between the marital status groups (F3,36 = 0.36, P = 0.781), suggesting that marital status did not have a relevant impact on the EAE.

Regarding GE Task 1, no significant differences were found (F3,36 = 0.67, P = 0.571), which suggested that marital status did not affect overall performance in the first task. The same occurred with IAE Task 1, where no significant differences were observed (F3,36 = 0.67, P = 0.571), indicating that the IAE in that task were not influenced by marital status. In EAE Task 1, a significant difference was found (F3,36 = 26.79, P < 0.011). Single participants committed fewer EAEs on this task compared to the other groups. Similarly, in IAE Task 2, a significant difference was found (F3,36 = 26.79, P < 0.011), with Singles showing a lower amount of IAE compared to the other groups. Regarding Task 2 EAEs, there were no significant differences (F3,36 = 0.39, P = 0.761), suggesting that marital status did not affect EAEs in that task.

Regarding GE Task 3, a significant difference was recorded (F3,36 = 26.79, P < 0.011), which indicated that general errors in this task were more frequent in Single participants compared to the other groups. However, no significant differences were found in IAE Task 3 (F3,36 = 0.36, P = 0.781) or EAE Task 3 (F3,36 = 0.39, P = 0.761), which suggested that marital status did not have an effect on IAE or EAE in the latter task.

In relation to the work situation (supplementary material 4 in S2 File), the comparison between active individuals (N=4) with inactive individuals (N=36) showed significant differences for some tasks and not for others. Regarding Total GA, a significant difference was observed between the active and inactive groups (F1,38 = 10.86, P < 0.013), indicating that active participants made fewer overall errors compared to inactive participants. For Total IAE, no significant differences were found between groups (F1,38 = 0.32, P = 0.573), suggesting that work status did not influence IAE. For Total EAEs, no significant differences were observed (F1,38 = 0.00, P = 1.003), indicating that employment status did not affect EAEs. Regarding EG Task 1, IAE Task 1 and EAE Task 1, no significant differences were found (F1,38 = 0.00, P = 1.003 for all variables), which indicates that the employment situation did not influence the GE or the IAE or EAE.

Regarding GE Task 2, no significant differences were found between the groups (F1,38 = 0.00, P = 1.003), while in IAE Task 2, no differences were also observed (F1,38 = 0.32, P = 0.573), suggesting that the work situation did not affect performance or IAE in this task. However, in EAE Task 2, a significant difference was found (F1,38 = 10.86, P < 0.013), indicating that active participants committed less EAE in this task compared to inactive participants.

Finally, in GE Task 3, IAE Task 3 and EAE Task 3, no significant differences were observed (F1,38 = 0.32, P = 0.573 for all variables), suggesting that the work situation did not influence the GE or the IAE or the EAE in this last task.

Table 2 presents the descriptive analyzes of the tasks in relation to the associated groups: Group 1 (Psychotics with hallucinations), Group 2 (Psychotics without hallucinations), Group 3 (Affective disorders) and Group 4 (Neurotic disorders).

Statistical analyzes showed that there were no significant differences between the groups in any of the variables evaluated. For the Total GE, the test value was F4,35 = 0.51, P = 0.731, which indicated that the diagnostic conditions did not influence the total GE of the participants. Similarly, for Total IAEs, the result was F4,35 = 0.09, P = 0.981, suggesting that there were no differences between the groups regarding IAEs. Regarding the Total EAEs, the value was F4,35 = 0.31, P = 0.871, which also indicated the absence of significant differences between the groups in the EAEs.

In the specific tasks (Task 1, Task 2 and Task 3) of GE, IAE and EAE, no significant differences were found between the groups. The values of the statistical tests for the different tasks were as follows: for GE Task 1, the value was F4,35 = 0.25, P = 0.911; for IAE Task 1, it was F4,35 = 0.25, P = 0.911; for EAE Task 1, F4,35 = 0.09, P = 0.981 was obtained. For GE Task 2, the value was F4,35 = 0.31, P = 0.871, for IAE Task 2, F4,35 = 0.09, P = 0.981; and for EAE Task 2, F4,35 = 0.51, P = 0.731. Finally, for GE Task 3, the result was F4,35 = 0.09, P = 0.981; for EAI Task 3, F4,35 = 0.31, P = 0.871; and for EAE Task 3, F4,35 = 0.51, P = 0.731.

The results of this study with the pencil-paper version (called TRP) [26] showed high specificity in psychotic patients, registering the highest average values of IAS errors among the four associated groups (Fig 1) and evidencing statistically significant differences (p=0.013) between psychotic patients with and without hallucinations (Table 3). These findings reinforce the value of the MMRT as a key tool in the assessment of source memory and reality monitoring, cognitive processes frequently altered in schizophrenia and fundamental for differentiating between experienced and imagined events.

**Table 3:** Descriptions of the tasks based on the group of Conditions.

	N	Group 1 (psychotics with hallucinations)	Group 2 (psychotic without hallucinations)	Group 3 (affective disorders)	Group 4 (neurotic disorders)	Test Statistic
		**(N=10)**	**(N=9)**	**(N=10)**	**(N=10)**	
GE Total	40	10.5 **11.0** 11.5	10.5 **11.0** 11.7	10.5 **11.0** 11.5	10.5 **11.0** 11.5	F_4,35_=0.51, P=0.73^1^
IAE Total	40	5.0 **5.3** 5.6	5.0 **5.3** 5.6	5.0 **5.3** 5.6	5.0 **5.3** 5.6	F_4,35_=0.09, P=0.98^1^
EAE Total	40	3.0 **3.4** 3.8	3.3 **3.8** 4.2	3.0 **3.4** 3.8	3.4 **3.8** 4.2	F_4,35_=0.31, P=0.87^1^
GE task 1	40	4.0 **4.1** 4.2	4.0 **4.2** 4.2	4.0 **4.1** 4.2	4.0 **4.1** 4.2	F_4,35_=0.25, P=0.91^1^
IAE Task 1	40	2.0 **2.0** 2.1	2.0 **2.1** 2.1	2.0 **2.0** 2.1	2.0 **2.0** 2.1	F_4,35_=0.25, P=0.91^1^
EAE Task 1	40	1.0 **1.1** 1.2	1.0 **1.1** 1.2	1.0 **1.1** 1.2	1.0 **1.1** 1.2	F_4,35_=0.09, P=0.98^1^
GE Task 2	40	3.0 **3.3** 3.6	3.2 **3.6** 3.9	3.0 **3.3** 3.6	3.3 **3.6** 3.9	F_4,35_=0.31, P=0.87^1^
IAE Task 2	40	1.0 **1.2** 1.4	1.0 **1.2** 1.4	1.0 **1.2** 1.4	1.0 **1.2** 1.4	F_4,35_=0.09, P=0.98^1^
EAE Task 2	40	1.6 **1.7** 1.8	1.6 **1.7** 1.8	1.6 **1.7** 1.8	1.6 **1.7** 1.8	F_4,35_=0.51, P=0.73^1^
GE Task 3	40	4.0 **4.4** 4.8	4.0 **4.4** 4.8	4.0 **4.4** 4.8	4.0 **4.4** 4.8	F_4,35_=0.09, P=0.98^1^
IAE Task 3	40	2.0 **2.2** 2.4	2.1 **2.4** 2.6	2.0 **2.2** 2.4	2.2 **2.4** 2.6	F_4,35_=0.31, P=0.87^1^
EAE Task 3	40	2.1 **2.4** 2.7	2.1 **2.4** 2.8	2.1 **2.4** 2.7	2.1 **2.4** 2.7	F_4,35_=0.51, P=0.73^1^

***Note:*** The table describes task performance across clinical groups, showing no significant differences.

Table 4 showed the correlations between the indicators of each subtask in GE, IAE and EAE. In GE, significant correlations were observed in all combinations of subtasks, with values of 0.38 (p=0.013) between 1 and 2, 0.45 (p=0.004) between 1 and 3, and 0.35 (p=0.0024) between 2 and 3, which indicated a consistent relationship between the indicators of this category.

**Table 4:** Relationship between the indicators of each subtask.

Error/subtask	1 y 2	1 y 3	2 y 3
GE	0,38 (p=0,013)	0,45 (p=0,004)	0,35 (p=0,0024)
IAE	0,27 (p=0,092)	0,45 (p=0,003)	0,14 (p=0,36)
EAE	0,43 (p=0,005)	0,54 (p=0,000)	0,34 (p=0,03)

***Note:*** The table shows significant correlations between the subtasks.

In IAE, the relationship between 1 and 2 presented a correlation of 0.27 with a p=0.092, which did not reach statistical significance. However, the correlation between 1 and 3 was 0.45 with p=0.003, which showed a significant association. On the other hand, the relationship between 2 and 3 had a coefficient of 0.14 with a p=0.36, indicating the absence of a statistically significant association in this combination. These results suggested that, within IAE, the connection between subtasks was less consistent compared to GE.

In EAE, all correlations were significant, with values of 0.43 (p=0.005) between 1 and 2, 0.54 (p=0.000) between 1 and 3, and 0.34 (p=0.03) between 2 and 3. The greatest association was evident between 1 and 3, reflecting a strong connection between these subtasks. The relationship between 2 and 3, although significant, showed the lowest coefficient within this group.

In general, the results indicated that GE and EAE presented more consistent correlations between the subtasks, while in IAE the associations were less uniform, with only one significant combination.

## Discusión

This research addressed the role of reality monitoring as a cognitive marker for the early detection of schizophrenia, leading to the development of a computer program for psychological assessment based on verbal tasks. The first findings in this field are documented in the research work of Johnson and Raye (1981) [8], who designed source memory tests with exploration of events that occurred in the past in which they demonstrated the existence of marked difficulty in differentiating between real and fictitious events in schizophrenics [31]. Through the measurement of a working memory task, memory fragmentation and poor sensory integration of reality in schizophrenia were demonstrated using fMRI [32].

A wide variety of experimental studies have tested similar tasks, which include at least one verbal component, such as a list of words in which the patient must remember the origin of the attribution, identifying whether the presented word was generated by the patient or by the examiner [16,21,23]. This pattern has also been observed in studies that have used MMRT-like verbal tasks in adolescents [33] and in schizophrenics with prefrontal cortex dysfunction [34], developmental impairment [35] or positive and/or negative symptoms [36,37,38].

## Combination of reality monitoring tasks-fMRI

Results of research similar to the one carried out by us or those carried out in combination with image resources highlight the importance of the software developed for the MMRT, since it will allow measuring the monitoring of reality memory in a more precise, effective, organized and ethical way. Additionally, the software programming includes the collection of sociodemographic and clinically relevant data, which facilitates the systematic identification and storage of information of patients undergoing this psychological evaluation.

The application of reality monitoring tasks, together with fMRI, indicate that cognitive deficits in schizophrenia may be related to various functional and structural disconnections in processing networks [23]. It has been observed that patients make more errors in the identification of self-referential elements [39], show low activation of the dorsolateral prefrontal cortex in working memory tasks [40], and show low activation of the anterior medial prefrontal cortex in reality monitoring tasks, when the information retrieved is related to internal or external aspects of the context [33,34].

## Incorporation of the BCT into the MMRT

Furthermore, as mentioned above, the MMRT is a test that includes a verbal component with the necessary characteristics to measure memory monitoring and has been developed from the theoretical model of Frith, Johnson and Raye. This allows its complementary use with the BCT test, a software that incorporates a visual and motor component, which has been patented by the Catholic University of Cuenca and is designed to evaluate the monitoring of current reality.

The combination of both tests would represent an innovative resource for the study of reality monitoring, due to the advanced technology they implement. This would allow accurate measurement of cognitive markers, facilitating early identification of schizophrenia and generating opportunities to improve patients’ quality of life by providing access to health services for early and timely interventions.

In future studies, the joint application of BCT and MMRT together with neuroimaging would allow us to examine the neural and behavioral responses during the execution of these tests. The use of techniques such as fMRI or Infrared Spectroscopy would provide detailed records on the activation of the prefrontal cortex (PFC), contributing to the validation of these instruments as early cognitive markers of Schizophrenia Spectrum Disorders, improving their quality of life [21].

## Benefits of the MMRT tool

MMRT software allows for easy program installation and the ability to perform the test from any modern computer. Additionally, the software offers multiple benefits such as language selection, audio, automatic typing, number of sessions, and training sessions that can be accessed to take the test multiple times. It also allows for systematic downloading of medical history data, informed consent, as well as the results of the number of times the test or cognitive training for long-term memory has been performed. As a complement, the program is easy to spread, since it only requires a link to be downloaded, installed and used following the instructions in the manual.

In the realm of cognitive and perceptual assessment, MMRT and BCT offer distinctive perspectives that are critical for a comprehensive understanding of schizophrenia spectrum disorders [41]. The MMRT, with its focus on verbal tasks, accurately examines an individual’s ability to distinguish between events that have been experienced and those that are imagined, assessing cognitive competencies associated with memory and introspection. This tool focuses on higher cognitive processing, especially in areas such as source memory and reality tracking, skills that are often compromised in individuals with schizophrenia.

On the other hand, BCT addresses perceptual and motor competencies through its visual and motor component, providing a dynamic assessment of the individual’s ability to interact with stimuli in real time and adjust their behavior accordingly. This approach allows us to examine sensorimotor integration and executive functions, which may also be affected in schizophrenia, although in a different way than those assessed by the MMRT.

The simultaneous use of the two tools can be particularly revealing, since schizophrenia spectrum disorders usually involve both cognitive and perceptual impairment [42]. The ability to correlate cognitive function with perceptual and motor ability may provide stronger indicators for the early detection of schizophrenia, as well as facilitate interventions that address the multiple dimensions of this complex group of disorders [43].

## Results of the clinical application of MMRT

The MMRT is distinguished by its innovative approach in the evaluation of reality monitoring functions, focusing on the measurement of internal and external attribution errors, fundamental aspects for the diagnosis of psychosis. Its effectiveness has been demonstrated in the differentiation of psychotic patients, with auditory hallucinations and without auditory hallucinations, as well as in the identification of differences with patients with affective and neurotic disorders [26]. Furthermore, its reliability is reinforced by the use of non-parametric tests, which allows obtaining accurate results even when the variables do not follow a normal distribution.

Complementing the MMRT, the BCT assesses perceptual and motor skills, focusing on individuals’ ability to interact with stimuli in real time. This dynamic assessment looks at sensorimotor integration and executive functions, areas that are not addressed by the MMRT. The integration of MMRT and BCT offers a comprehensive understanding of schizophrenia spectrum disorders, allowing specialists to jointly evaluate cognitive, perceptual and motor skills, as well as understand their interrelationships. This dual assessment is crucial, given that schizophrenia is a multifaceted disorder, typically involving both cognitive and perceptual impairments [44]. The ability to correlate cognitive function with perceptual and motor skills could provide stronger indicators for early detection and intervention [26].

The results obtained in this research did not find statistically significant differences in “central monitoring” between the pathology groups considering that the test results focused on the global indicators of the task presented; on the contrary, the independent analysis of the three parts of the test showed differences in terms of “internal attribution errors” in subtask 1. In addition, when comparing the averages of groups two by two, differences were found in the pair: “psychotics with hallucinations”-”psychotics without hallucinations.” The non-existence of differences between the “psychotics with hallucinations” group and the rest of the groups could be related to unanalyzed variables that acted as sources of error. The “psychotics without hallucinations” group showed a minimal difference associated with common characteristics in relation to the “psychotics with hallucinations” group.

The results obtained show marked differences with previous research in relation to the period elapsed from when the subject generates the words until he is asked to recognize them [45] and in relation to the evaluation interval between the phases [46]. Furthermore, the degree of involvement of working memory does not appear to be stable and is mediated by a reduction in the waiting interval between word generation and presentation of the recognition list [47].

The exploration of the impact of external monitoring affected by delusional symptoms could be explored by including in the task two external stimuli demanding the subject to discriminate between them [48,49], constituting a new challenge to the analysis.

As can be understood from the results obtained, it should not be considered that memory processes are unrelated to the discrimination functions of the stimulus source. Therefore, the imposition of restrictions, for example, of an orthographic type, on patients with “auditory hallucinations” are especially affected and impoverish their performance compared to that of other psychotic patients [50,51]. This may occur either because their semantic repertoire is really idiosyncratic and when it is reduced by the demands of the task, decisive clues are lost for them, or because due to these restrictions they follow false stimulus cues, different from those of other subjects [51–53]. For this reason, if “psychotic patients with auditory hallucinations” present special difficulties in this subtask, it must be assumed that it is due to the characteristics of the task [8].

The use of Reality Monitoring (MR) in other specialties has been demonstrated in recent studies. For example, a meta-analytic review on the validity of this tool in the forensic field, found a total score in the RM discriminated, d = 0.542 (δ = 0.562), between memories of imagined and perceived events, where the results supported the predictions of the model (more external attributes in perceived memories) in the criteria clarity, d = 0.361 (δ = 0.399), sensory information, d = 0.359 (δ = 0.397), spatial information, d = 0.250 (δ = 0.277), temporal information, d = 0.509 (δ = 0.563), story reconstruction, d = 0.441 (δ = 0.488), and realism, d = 0.420 (δ = 0.464), but not for the affective information criterion, d = 0.024 [-0.081, 0.129] [41].

Some moderators of the effects such as age (more cognitive operations in imagined memories in adults and in perceived memories in minors), type of evocation (external attributes discern between imagined and perceived memories, both in self-experienced and non-experienced stories) and the criterion score (the results differed according to the criterion score) have contributed to the implications of the results regarding the validity of RM as a forensic technique in judicial procedures [41].

## Conclusion

MMRT alone or combined with other tests such as BCT is a useful and friendly tool for the early detection of attribution errors in subjects at risk of suffering from schizophrenia and in the detection of positive symptoms of this disease. The objective of this study is specified with an adequate formalization of cognitive markers such as MMRT and BCT, becoming reference software for modeling schizophrenia spectrum disorders as disorders with a clear and precise cognitive component around reality monitoring and memory monitoring. This tool offers an advance in the confirmation of the detection of hallucinations in schizophrenic or at-risk subjects, generating a considerable impact on the diagnostic confirmation of the most important symptom in episodes of psychiatric decompensation. The complementary approach of these tools offers a holistic view of the cognitive-perceptual deficits characteristic of this health condition, facilitating targeted interventions. Establishing the adequacy of cognitive models to symptomatic functioning in schizophrenia spectrum disorders is essential to understanding the disease. The results obtained support the reliability and specificity of these instruments. Thus, its value in clinical neuropsychiatry is highlighted, in line with new transdiagnostic approaches and the expansion of indicators that support clinical judgment [26].

## Supporting information

S1 Fig MMRT main screen. Illustrates the MMRT home screen, highlighting training, login, setup, and user management options, along with a tutorial player.
(TIF)

S2 Fig MMRT general settings. Presents the MMRT settings section, with options to set words in training mode and enable voice accessibility, along with buttons to save or return.
(TIF)

S3 Fig MMRT admin settings. Displays the MMRT administrative configuration screen, with functions for listing and adding users, downloading data, and managing sessions, as well as return or exit buttons.
(TIF)

S4 Fig MMRT Main test screen. Represents the MMRT testing interface, where (a) it allows the selection of users, (b) it asks the user to write a semantically related word, and (c) it requires choosing who the word belongs to between two options, computer or user.
(TIF)

S5 Fig MMRT workflow. Fig S5 shows the MMRT workflow, starting from the home page and progressing through session and user verification, to performing and recording test results.
(TIFF)

S6 Fig Voice recognition workflow. Fig S6 details the speech recognition workflow in MMRT, ranging from activating instructions to collecting and converting user voice responses, ending with saving results.
(TIFF)

S1 File This file contains the supplementary tables. (XLSX)

S2 File Supplementary file. (DOCX)
