# Supplementary figures and images for: Memory Monitoring Recognition Test (MMRT), a new measurement of stimular source monitoring: Software and comprehension
Source: PLoS One. 2025 Apr 28;20(4):e0321991. doi: 10.1371/journal.pone.0321991 (PMC12036938; doi:10.1371/journal.pone.0321991)

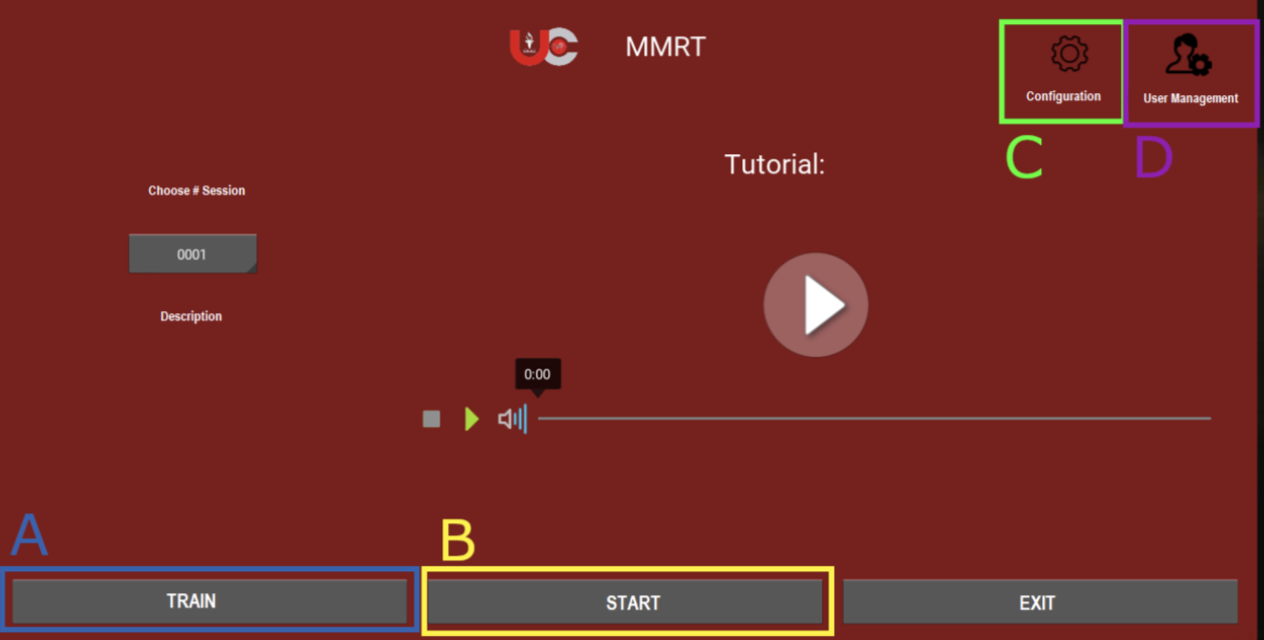

Supplement: S1 Fig — Illustrates the MMRT home screen, highlighting training, login, setup, and user management options, along with a tutorial player. (TIF) [file pone.0321991.s001.tif]

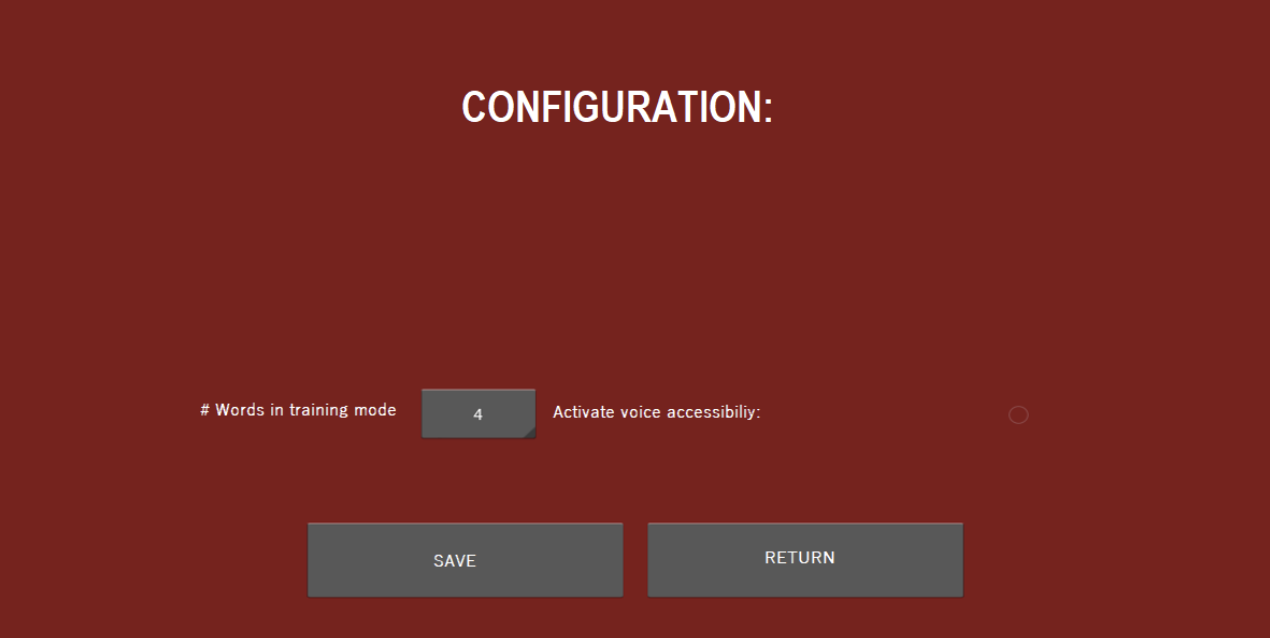

Supplement: S2 Fig — Presents the MMRT settings section, with options to set words in training mode and enable voice accessibility, along with buttons to save or return. (TIF) [file pone.0321991.s002.tif]

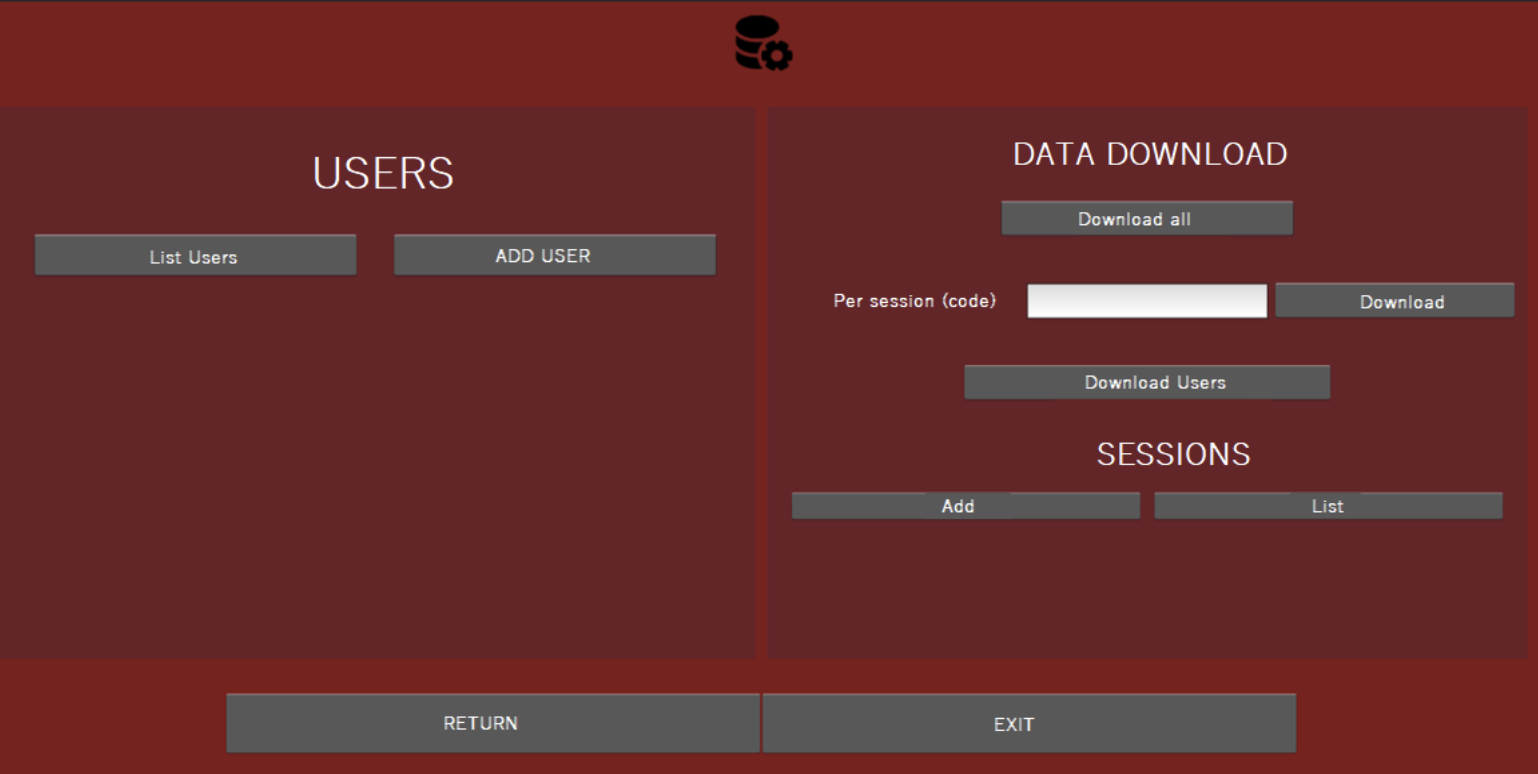

Supplement: S3 Fig — Displays the MMRT administrative configuration screen, with functions for listing and adding users, downloading data, and managing sessions, as well as return or exit buttons. (TIF) [file pone.0321991.s003.tif]

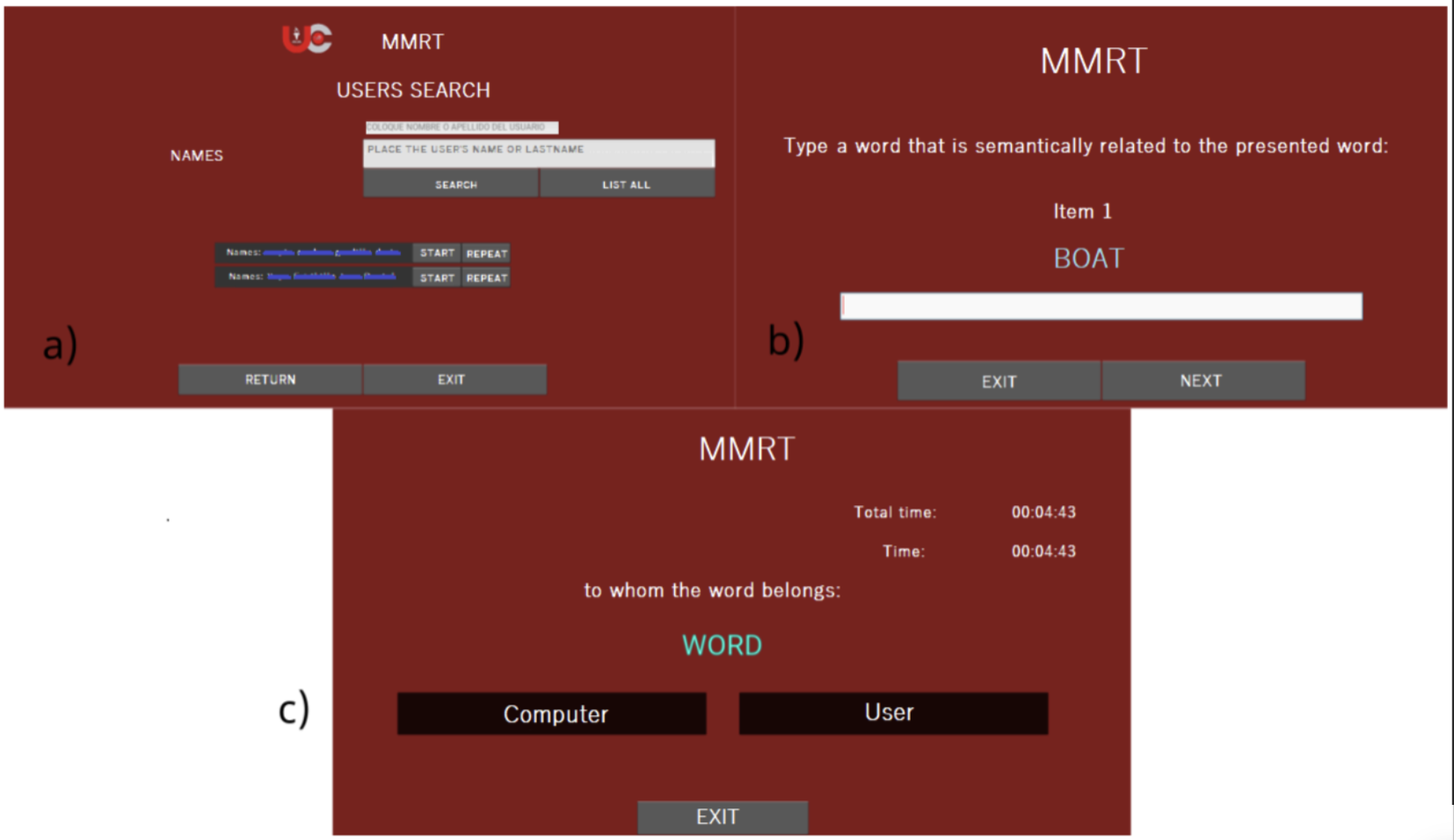

Supplement: S4 Fig — Represents the MMRT testing interface, where (a) it allows the selection of users, (b) it asks the user to write a semantically related word, and (c) it requires choosing who the word belongs to between two options, computer or user. (TIF) [file pone.0321991.s004.tif]

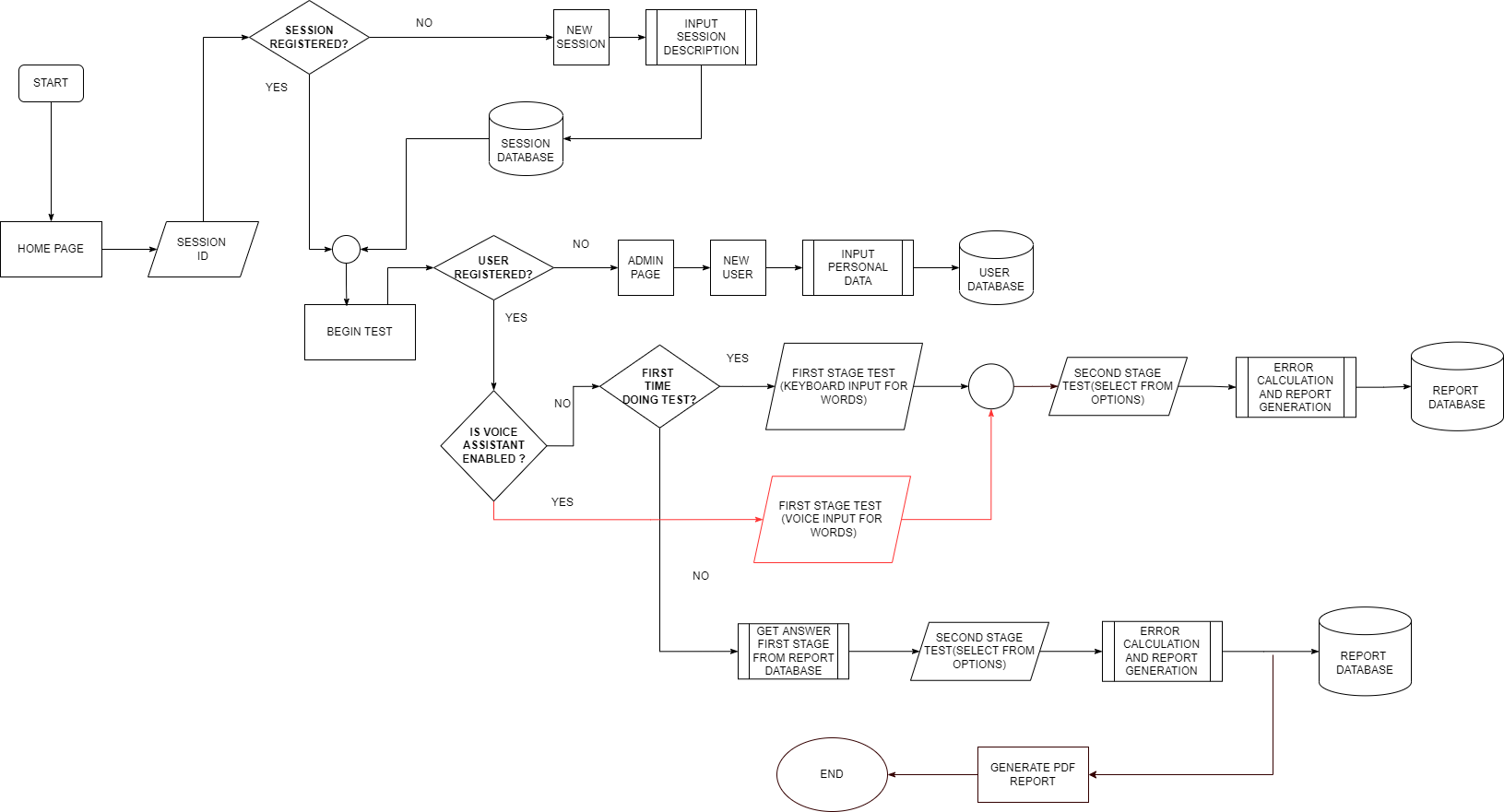

Supplement: S5 Fig — Fig S5 shows the MMRT workflow, starting from the home page and progressing through session and user verification, to performing and recording test results. (TIFF) [file pone.0321991.s005.tiff]

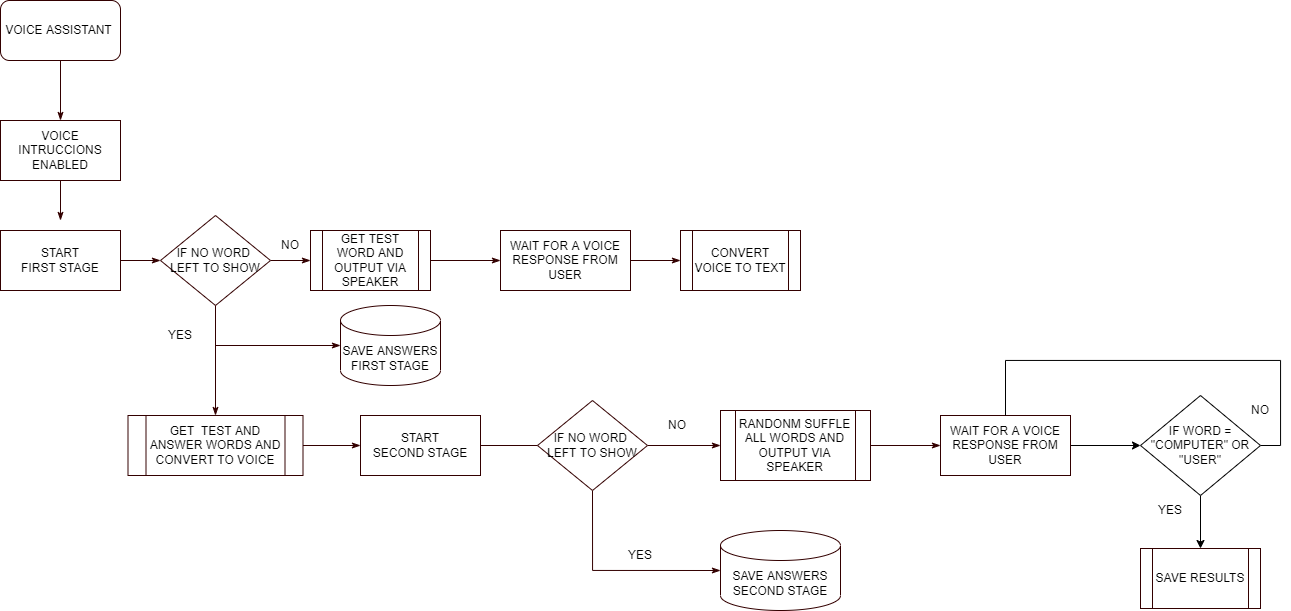

Supplement: S6 Fig — Fig S6 details the speech recognition workflow in MMRT, ranging from activating instructions to collecting and converting user voice responses, ending with saving results. (TIFF) [file pone.0321991.s006.tiff]
