# Supplementary material for: Memory Monitoring Recognition Test (MMRT), a new measurement of stimular source monitoring: Software and comprehension
Source: PLoS One. 2025 Apr 28;20(4):e0321991. doi: 10.1371/journal.pone.0321991 (PMC12036938; doi:10.1371/journal.pone.0321991)
Supplement: S1 File — (XLSX) [file pone.0321991.s007.docx]

**Supplementary material**

**Supplementary material 1.** Descriptions of the tasks according to sex and age group

|  | **N** | **Woman** | **Man** | **Test Statistic** | **18-19 years** | **20-22 years** | **Test Statistic** |
| --- | --- | --- | --- | --- | --- | --- | --- |
|  |  | **(N=27)** | **(N=13)** |  | **(N=16)** | **(N=24)** |  |
| GE  Total | 40 | 10.5 **11.0** 11.5 | 10.3 **11.0** 11.7 | F_1,38_=0.00, P=1.00^3^ | 10.0 **10.2** 10.5 | 11.0 **11.5** 12.0 | F_1,38_=114.00, P<0.01^3^ |
| IAE  Total | 40 | 5.0 **5.3** 5.6 | 5.0 **5.3** 5.6 | F_1,38_=0.07, P=0.79^3^ | 5.0 **5.3** 5.6 | 5.0 **5.3** 5.6 | F_1,38_=0.05, P=0.82^3^ |
| EAE  Total | 40 | 3.4 **3.8** 4.2 | 3.0 **3.0** 3.8 | F_1,38_=5.59, P=0.02^3^ | 3.2 **3.6** 4.0 | 3.2 **3.6** 4.0 | F_1,38_=0.00, P=1.00^3^ |
| GE  task 1 | 40 | 4.0 **4.2** 4.2 | 4.0 **4.0** 4.0 | F_1,38_=35.29, P<0.01^3^ | 4.0 **4.1** 4.2 | 4.0 **4.1** 4.2 | F_1,38_=0.00, P=1.00^3^ |
| IAE  task 1 | 40 | 2.0 **2.1** 2.1 | 2.0 **2.0** 2.0 | F_1,38_=35.29, P<0.01^3^ | 2.0 **2.0** 2.1 | 2.0 **2.0** 2.1 | F_1,38_=0.00, P=1.00^3^ |
| EAE  task 1 | 40 | 1.0 **1.1** 1.2 | 1.0 **1.1** 1.2 | F_1,38_=0.07, P=0.79^3^ | 1.0 **1.1** 1.2 | 1.0 **1.1** 1.2 | F_1,38_=0.05, P=0.82^3^ |
| GE  task 2 | 40 | 3.3 **3.6** 3.9 | 3.0 **3.0** 3.6 | F_1,38_=5.59, P=0.02^3^ | 3.1 **3.5** 3.8 | 3.1 **3.5** 3.8 | F_1,38_=0.00, P=1.00^3^ |
| IAE  ask 2 | 40 | 1.0 **1.2** 1.4 | 1.0 **1.2** 1.4 | F_1,38_=0.07, P=0.79^3^ | 1.0 **1.2** 1.4 | 1.0 **1.2** 1.4 | F_1,38_=0.05, P=0.82^3^ |
| EAE  task 2 | 40 | 1.6 **1.7** 1.8 | 1.6 **1.7** 1.8 | F_1,38_=0.00, P=1.00^3^ | 1.5 **1.6** 1.6 | 1.7 **1.8** 1.9 | F_1,38_=114.00, P<0.01^3^ |
| GE  task 3 | 40 | 4.0 **4.4** 4.8 | 4.0 **4.4** 4.8 | F_1,38_=0.07, P=0.79^3^ | 4.0 **4.4** 4.8 | 4.0 **4.4** 4.8 | F_1,38_=0.05, P=0.82^3^ |
| IAE  ask 3 | 40 | 2.2 **2.4** 2.6 | 2.0 **2.0** 2.4 | F_1,38_=5.59, P=0.02^3^ | 2.1 **2.3** 2.5 | 2.1 **2.3** 2.5 | F_1,38_=0.00, P=1.00^3^ |
| EAE  task 3 | 40 | 2.1 **2.4** 2.7 | 2.0 **2.4** 2.8 | F_1,38_=0.00, P=1.00^3^ | 1.8 **2.0** 2.1 | 2.4 **2.7** 3.0 | F_1,38_=114.00, P<0.01^3^ |
| ***Note:*** ^3^Wilcoxon. IAE = internal attribution errors, EAE = external  attribution errors. | | | | |  |  |  |

**Supplementary material 2.** Descriptions of the tasks depending on the educational level

|  | N | Bachelor | Primary | Without studies | University | Test Statistic |
| --- | --- | --- | --- | --- | --- | --- |
|  |  | **(N=16)** | **(N=17)** | **(N=4)** | **(N=3)** |  |
| GE  Total | 40 | 10.5 **11.2** 11.8 | 10.3 **11.0** 11.5 | 10.4 **11.2** 11.8 | 10.5 **10.5** 11.8 | F_3,36_=0.42, P=0.74^1^ |
| IAE  Total | 40 | 5.0 **5.0** 5.3 | 5.2 **5.3** 5.6 | 5.3 **5.4** 5.6 | 5.3 **5.6** 5.6 | F_3,36_=3.19, P=0.04^1^ |
| EAE  Total | 40 | 3.4 **3.8** 4.2 | 3.0 **3.0** 3.5 | 3.6 **3.8** 4.0 | 3.5 **3.8** 4.1 | F_3,36_=5.65, P<0.01^1^ |
| GE  task 1 | 40 | 4.0 **4.2** 4.2 | 4.0 **4.0** 4.2 | 4.0 **4.1** 4.2 | 4.0 **4.2** 4.2 | F_3,36_=1.90, P=0.15^1^ |
| IAE  task 1 | 40 | 2.0 **2.1** 2.1 | 2.0 **2.0** 2.1 | 2.0 **2.0** 2.1 | 2.0 **2.1** 2.1 | F_3,36_=1.90, P=0.15^1^ |
| EAE  task 1 | 40 | 1.0 **1.0** 1.1 | 1.1 **1.1** 1.2 | 1.1 **1.2** 1.2 | 1.1 **1.2** 1.2 | F_3,36_=3.19, P=0.04^1^ |
| GE  task 2 | 40 | 3.3 **3.6** 3.9 | 3.0 **3.0** 3.4 | 3.4 **3.6** 3.8 | 3.4 **3.6** 3.8 | F_3,36_=5.65, P<0.01^1^ |
| IAE  task 2 | 40 | 1.0 **1.0** 1.2 | 1.1 **1.2** 1.4 | 1.2 **1.3** 1.4 | 1.2 **1.4** 1.4 | F_3,36_=3.19, P=0.04^1^ |
| EAE  task 2 | 40 | 1.6 **1.8** 1.9 | 1.6 **1.7** 1.8 | 1.6 **1.8** 1.9 | 1.6 **1.6** 1.8 | F_3,36_=0.42, P=0.74^1^ |
| GE  task 3 | 40 | 4.0 **4.0** 4.4 | 4.3 **4.4** 4.8 | 4.4 **4.6** 4.8 | 4.5 **4.8** 4.8 | F_3,36_=3.19, P=0.04^1^ |
| IAE  task 3 | 40 | 2.2 **2.4** 2.6 | 2.0 **2.0** 2.3 | 2.3 **2.4** 2.5 | 2.2 **2.4** 2.6 | F_3,36_=5.65, P<0.01^1^ |
| EAE  task 3 | 40 | 2.1 **2.6** 2.9 | 2.0 **2.4** 2.7 | 2.0 **2.5** 2.9 | 2.1 **2.1** 2.8 | F_3,36_=0.42, P=0.74^1^ |
| ***Note:*** ^1^Kruskal-Wallis. | | | |  |  |  |

**Supplementary material 3.** Task descriptions based on marital status

|  | **N** | **Married** | **Separated or divorced** | **Single** | **Widower** | **Test Statistic** |
| --- | --- | --- | --- | --- | --- | --- |
|  |  | **(N=19)** | **(N=6)** | **(N=13)** | **(N=2)** |  |
| GE Total | 40 | 10.5 **11.0** 11.5 | 10.9 **11.2** 12.0 | 10.3 **11.0** 11.5 | 10.5 **11.2** 12.0 | F_3,36_=0.39, P=0.76^1^ |
| IAE Total | 40 | 5.3 **5.3** 5.6 | 5.3 **5.4** 5.6 | 5.0 **5.0** 5.0 | 5.3 **5.4** 5.6 | F_3,36_=26.79, P<0.01^1^ |
| EAE Total | 40 | 3.1 **3.4** 3.8 | 3.4 **3.8** 4.2 | 3.0 **3.4** 3.9 | 3.4 **3.8** 4.2 | F_3,36_=0.36, P=0.78^1^ |
| GE task 1 | 40 | 4.0 **4.0** 4.2 | 4.0 **4.1** 4.2 | 4.0 **4.0** 4.2 | 4.2 **4.2** 4.2 | F_3,36_=0.67, P=0.57^1^ |
| IAE task 1 | 40 | 2.0 **2.0** 2.1 | 2.0 **2.0** 2.1 | 2.0 **2.0** 2.1 | 2.1 **2.1** 2.1 | F_3,36_=0.67, P=0.57^1^ |
| EAE task 1 | 40 | 1.1 **1.1** 1.2 | 1.1 **1.2** 1.2 | 1.0 **1.0** 1.0 | 1.1 **1.1** 1.2 | F_3,36_=26.79, P<0.01^1^ |
| GE task 2 | 40 | 3.0 **3.3** 3.6 | 3.3 **3.6** 3.9 | 3.0 **3.3** 3.7 | 3.3 **3.6** 3.9 | F_3,36_=0.36, P=0.78^1^ |
| IAE task 2 | 40 | 1.2 **1.2** 1.4 | 1.2 **1.3** 1.4 | 1.0 **1.0** 1.0 | 1.2 **1.3** 1.4 | F_3,36_=26.79, P<0.01^1^ |
| EAE task 2 | 40 | 1.6 **1.7** 1.8 | 1.7 **1.8** 1.9 | 1.6 **1.7** 1.8 | 1.6 **1.8** 1.9 | F_3,36_=0.39, P=0.76^1^ |
| GE task 3 | 40 | 4.4 **4.4** 4.8 | 4.4 **4.6** 4.8 | 4.0 **4.0** 4.0 | 4.4 **4.6** 4.8 | F_3,36_=26.79, P<0.01^1^ |
| IAE task 3 | 40 | 2.0 **2.2** 2.4 | 2.2 **2.4** 2.6 | 2.0 **2.2** 2.5 | 2.2 **2.4** 2.6 | F_3,36_=0.36, P=0.78^1^ |
| EAE task 3 | 40 | 2.1 **2.4** 2.7 | 2.4 **2.5** 3.0 | 2.0 **2.4** 2.7 | 2.1 **2.5** 3.0 | F_3,36_=0.39, P=0.76^1^ |
| ***Note:*** ^1^Kruskal-Wallis. | | | |  |  |  |

**Supplementary material 4.** Descriptions of the tasks depending on the work situation

|  | **N** | **Activo** | **Inactivo** | **Test Statistic** |
| --- | --- | --- | --- | --- |
|  |  | **(N=4)** | **(N=36)** |  |
| GE Total | 40 | 10.0 **10.0** 10.0 | 10.5 **11.0** 11.5 | F_1,38_=10.86, P<0.01^3^ |
| IAE Total | 40 | 5.0 **5.2** 5.5 | 5.0 **5.3** 5.6 | F_1,38_=0.32, P=0.57^3^ |
| EAE Total | 40 | 3.2 **3.6** 4.0 | 3.2 **3.6** 4.0 | F_1,38_=0.00, P=1.00^3^ |
| GE task 1 | 40 | 4.0 **4.1** 4.2 | 4.0 **4.1** 4.2 | F_1,38_=0.00, P=1.00^3^ |
| IAE task 1 | 40 | 2.0 **2.0** 2.1 | 2.0 **2.0** 2.1 | F_1,38_=0.00, P=1.00^3^ |
| EAE task 1 | 40 | 1.0 **1.0** 1.2 | 1.0 **1.1** 1.2 | F_1,38_=0.32, P=0.57^3^ |
| GE task 2 | 40 | 3.1 **3.4** 3.8 | 3.1 **3.4** 3.8 | F_1,38_=0.00, P=1.00^3^ |
| IAE task 2 | 40 | 1.0 **1.1** 1.3 | 1.0 **1.2** 1.4 | F_1,38_=0.32, P=0.57^3^ |
| EAE task 2 | 40 | 1.5 **1.5** 1.5 | 1.6 **1.7** 1.8 | F_1,38_=10.86, P<0.01^3^ |
| GE task 3 | 40 | 4.0 **4.2** 4.6 | 4.0 **4.4** 4.8 | F_1,38_=0.32, P=0.57^3^ |
| IAE task 3 | 40 | 2.1 **2.3** 2.5 | 2.1 **2.3** 2.5 | F_1,38_=0.00, P=1.00^3^ |
| EAE task 3 | 40 | 1.8 **1.8** 1.8 | 2.1 **2.4** 2.7 | F_1,38_=10.86, P<0.01^3^ |
| ***Note:***  ^3^Wilcoxon. | | | |  |
